# Construction of a novel quinoxaline as a new class of Nrf2 activator

**DOI:** 10.1186/s13065-019-0633-4

**Published:** 2019-09-24

**Authors:** Murugesh Kandasamy, Kit-Kay Mak, Thangaraj Devadoss, Punniyakoti Veeraveedu Thanikachalam, Raghavendra Sakirolla, Hira Choudhury, Mallikarjuna Rao Pichika

**Affiliations:** 1 Department of Pharmaceutical Chemistry, School of Pharmacy, International Medical University, Kuala Lumpur, Malaysia; 2 School of Postgraduate Studies and Research, International Medical University, Kuala Lumpur, Malaysia; 3 Department of Pharmaceutical Technology, School of Pharmacy, International Medical University, Kuala Lumpur, Malaysia; 4 Center for Bioactive Molecules & Drug Delivery, Institute for Research, Development & Innovation, International Medical University, Kuala Lumpur, Malaysia; 5 KVSR Siddhartha College of Pharmaceutical Sciences, Vijayawada, Andhra Pradesh India; 6 ISF College of Pharmacy, Moga, Punjab India; 7 Department of Chemistry, Central University of Karnataka, Gulbarga, Kanataka India

**Keywords:** *N*′-nicotinoylquinoxaline-2-carbohydrazide, NRF2, KEAP1, Anti-inflammatory, Metabolic stability, Molecular docking

## Abstract

**Background:**

The transcription factor Nuclear factor erythroid-2-related factor 2 (NRF2) and its principal repressive regulator, Kelch-like ECH-associated protein 1 (KEAP1), are perilous in the regulation of inflammation, as well as maintenance of homeostasis. Thus, NRF2 activation is involved in cytoprotection against many inflammatory disorders. *N*′-Nicotinoylquinoxaline-2-carbohdyrazide (NQC) was structurally designed by the combination of important pharmacophoric features of bioactive compounds reported in the literature.

**Methods:**

NQC was synthesised and characterised using spectroscopic techniques. The compound was tested for its anti-inflammatory effect using Lipopolysaccharide from *Escherichia coli* (LPS*Ec*) induced inflammation in mouse macrophages (RAW 264.7 cells). The effect of NQC on inflammatory cytokines was measured using enzyme-linked immune sorbent assay (ELISA). The Nrf2 activity of the compound NQC was determined using ‘Keap1:Nrf2 Inhibitor Screening Assay Kit’. To obtain the insights on NQC’s activity on Nrf2, molecular docking studies were performed using Schrödinger suite. The metabolic stability of NQC was determined using mouse, rat and human microsomes.

**Results:**

NQC was found to be non-toxic at the dose of 50 µM on RAW 264.7 cells. NQC showed potent anti-inflammatory effect in an in vitro model of LPS*Ec* stimulated murine macrophages (RAW 264.7 cells) with an IC_50_ value 26.13 ± 1.17 µM. NQC dose-dependently down-regulated the pro-inflammatory cytokines [interleukin (IL)-1β (13.27 ± 2.37 μM), IL-6 (10.13 ± 0.58 μM) and tumor necrosis factor (TNF)-α] (14.41 ± 1.83 μM); and inflammatory mediator, prostaglandin E_2_ (PGE_2_) with IC_50_ values, 15.23 ± 0.91 µM. Molecular docking studies confirmed the favourable binding of NQC at Kelch domain of Keap-1. It disrupts the Nrf2 interaction with kelch domain of keap 1 and its IC_50_ value was 4.21 ± 0.89 µM. The metabolic stability studies of NQC in human, rat and mouse liver microsomes revealed that it is quite stable with half-life values; 63.30 ± 1.73, 52.23 ± 0.81, 24.55 ± 1.13 min; microsomal intrinsic clearance values; 1.14 ± 0.31, 1.39 ± 0.87 and 2.96 ± 0.34 µL/min/g liver; respectively. It is observed that rat has comparable metabolic profile with human, thus, rat could be used as an in vivo model for prediction of pharmacokinetics and metabolism profiles of NQC in human.

**Conclusion:**

NQC is a new class of NRF2 activator with potent in vitro anti-inflammatory activity and good metabolic stability.

## Introduction

Lead generation is one of the key challenge in drug discovery and development process, and it is the search of chemical compounds that will be therapeutically effective against a disease. Fragment-based drug discovery (FBDD) is a technique often used to generate structures known as ‘leads’ [1]. From the literature search, a number of key structures were discovered to be potent anti-inflammatory agents. This includes quinoxaline [2], hydrazine [3] and pyridine. Thus, in this study, we aim to synthesised these fragments into a lead and to investigate its anti-inflammatory activity via Nrf2 activation.

**Fig. 1:**
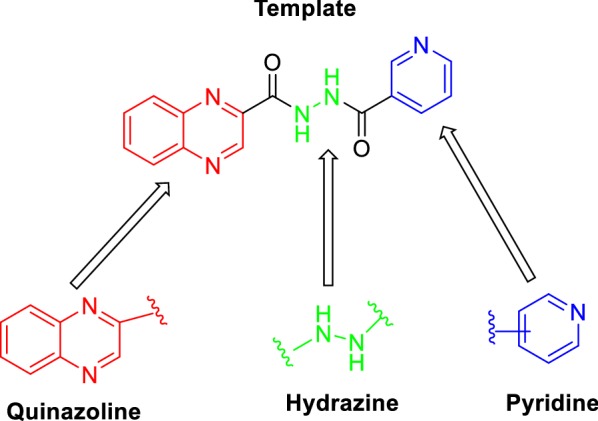
Construction of template

Quinoxaline, fused ring of benzene and pyrazine, is one of the important class of heterocyclic compound having diverse biological activities [4–10] and present as integral part of diverse bioactive compounds and pharmaceuticals [11–15]. Hydrazines are nitrogen–nitrogen bond containing compounds which exhibit remarkable biological activities [16–19]. Pyridines possess many biological activities and present as an integral part of many medicinal compounds [20]. Quinoxalines substituted at second position are reported to possess remarkable biological activities [21]. Therefore, combining quinoxaline, hydrazine and pyridine moieties into one molecule as represented in Fig. 1 was believed to be a potential template for the synthesis of novel class of bioactive compounds.

**Fig. 2:**
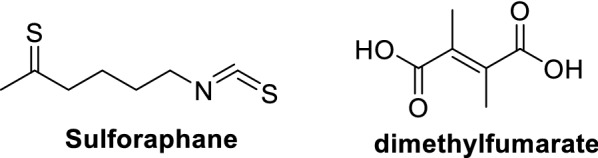
Structures of Nrf2 activators

Many reports in the literature demonstrated Nuclear factor erythroid 2 -related factor 2 (Nrf2) activation contributes to diverse biological activities by regulating the Kelch-like ECH-associated protein (Keap1)/Nrf2 signaling pathway [22–24]. Therefore Nrf2 activation has emerged as an attractive therapeutic approach to develop new classes of drugs as therapeutic treatment for a myriad of diseases and this includes inflammation, chronic multiple sclerosis, kidney disease, pulmonary fibrosis, cancer and chronic obstructive pulmonary disease (COPD) [25–32]. In literature, two well-studied Nrf2 activators were reported to be sulforaphane (a derived isothiocyanate from broccoli; and dimethyl fumarate, a new drug for the treatment of multiple sclerosis (Fig. 2). However, these two produce side effects due to the presence of strong electrophilic functional groups and hence covalently react with other proteins [33].

**Fig. 3:**
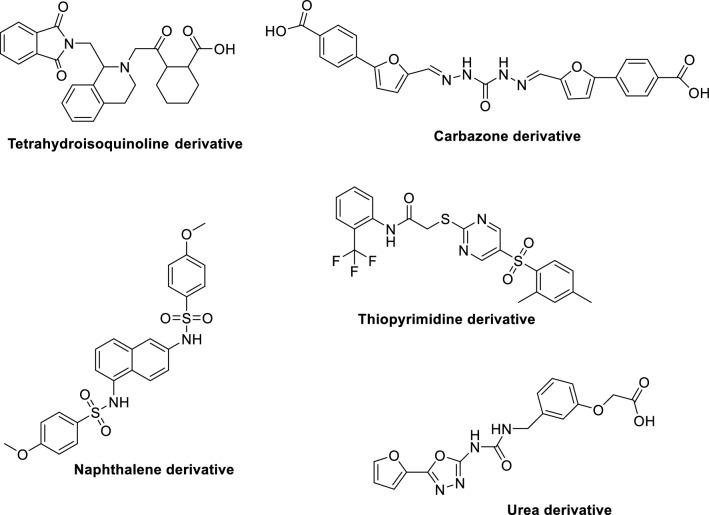
Reported Nrf2 activators

Another natural Nrf2 activator is oleanic triterpenoid bardoxolone imidazole. It was withdrawn from phase III clinical trials for patients diagnosed with type 2 diabetes and chronic kidney disease because of adverse cardiovascular events [34] that might be due to its covalent binding with key residues in protein. From these observations, it is postulated that the development of reversible covalent and non-covalent Nrf2 activators would be an ideal strategy to develop selective and safe Nrf2 activators [24, 35–38]. The above said efforts resulted in five series of compounds; tetrahydroisoquinoline, carbazone, naphthalene, thiopyrimidine and urea derivatives; that shown to activate Nrf2 whose representative structures were shown in Fig. 3.

It is noted that the proposed template and reported Nrf2 activators have few structural similarities. Therefore, in this study we synthesised the template compound, tested its efficacy in reversing the nitric oxide (NO) production, levels of pro-inflammatory cytokines (IL-1β, IL-6 and TNF-α), level of inflammatory mediator (Prostaglandin E_2_ (PGE_2_)) in LPS stimulated RAW 264.7 cells. Its’ efficacy in disrupting the interaction between kelch domain of Keap 1 and Nrf2 was determined and in silico molecular docking studies were performed to acquire insights on its molecular interactions at the interface of Nrf2/Keap1. The metabolic stability profile of the compound was determined using liver microsomes (human, rat and mouse). We thought of proceeding with the synthesis of analogues only if the template compound showed promising anti-inflammatory and metabolic stability and therefore, the analogues were not synthesised.

## Materials and methods

### General

*N*′-(Pyridine-3-carbonyl)quinoxaline-2-carbohydrazide (NQC) was synthesised and purified to 99.2% purity in International Medical University (IMU). Structure of the quinoxaline-2-carbohydrazide derivative was confirmed by spectroscopic methods. All solvents used in this research were of high performance column chromatography (HPLC) grade. Chemical reagents including formic acid and dipotassium phosphate (K_2_HPO_4_) from Fisher Scientific, dimethyl sulfoxide (DMSO) and monopotassium phosphate (KH_2_PO_4_) from Merck, and acetonitrile (ACN) from Friedemann Schmidt were used. (3-(4,5-Dimethylthiazol-2-yl)-2,5-Diphenyltetrazolium Bromide), sulphanilamide, *N*-(1-naphthyl)ethylenediamine and biological grade DMSO were procured from Sigma-Aldrich Sdn. Bhd, Malaysia. β-NADPH was procured from Sigma Aldrich, USA. RAW 264.7 cells and LPS (*Escherichia coli* O111:b4) were procured from American Type Culture Collection, Manassas, USA. HLM, RLM and MLM (20 mg/mL, Catalog #HMMCPL, Gibco) was procured from Life Technologies, Singapore.

**Fig. 4:**
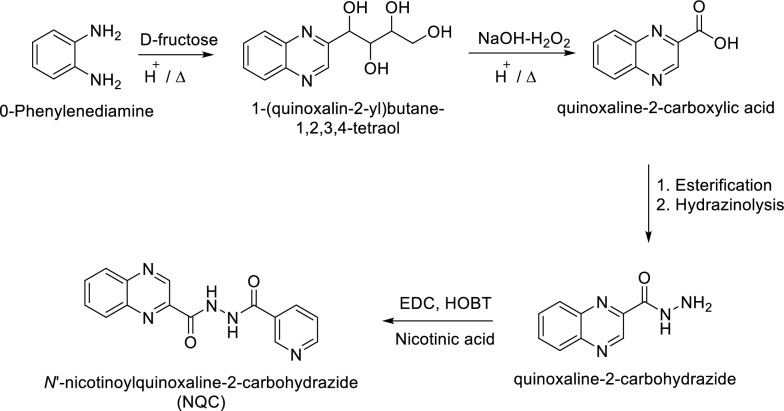
Synthetic route of *N*′-(pyridine-3-carbonyl) quinoxaline-2-carbohydrazide (NQC)

Synthesis of *N*′-(pyridine-3-carbonyl)quinoxaline-2-carbohydrazide is as shown in Fig. 4.

### Cell culture

Murine macrophages, cell line: RAW 264.7 was procured from ATCC, USA. The macrophage cells were grown and cultured in Dulbecco’s Modified Eagle Medium (DMEM) supplemented with 10% Fetal Bovine serum (FBS) and 1% PenStrep and incubated in a 5% CO_2_ at 37 °C incubator. Cells from passages 10 to 20 were used for subsequent experiments.

### Cell viability

A calorimetric agent (3-(4,5-Dimethylthiazol-2-yl)-2,5-Diphenyltetrazolium Bromide) (MTT) was used to as an assay to determine cell viability after being exposed to NQC. RAW 264.7 cells were seeded in a 96-well microplate at 3 × 10^5^ cells/well and were allowed to grow and adhere for 24 h in a 37 °C, 5% CO_2_ incubator. NQC was dissolved in DMSO and diluted with PBS to produce the required test concentrations (100, 50, 25, 12.5, 6.25 and 3.125 µM). The negative control is 0.1% (v/v) DMSO, which is the maximum effective concentration of DMSO in a well. The cells were treated with either NQC or 0.1% (v/v) DMSO for 4 h followed by 1 µg/mL LPS*Ec* and incubated further for 20 h. MTT solution was added at 100 µL per well under dark condition and the cells were incubated further for 4 h. Upon completion of incubation, the contents in all wells were aspirated and DMSO was added at 100 µL per well. The absorbance was measured at 570 nm using Molecular Devices Spectramax M3 Multi-Mode microplate reader; Sunnyvale, CA, USA. The cells without any treatment was used as a control. MTT assay was performed in triplicate. The percent cell viability is calculated using the following equation:

$$ Percent \;Cell\; Viabilty = \frac{Absorbance \;of\; Vehicle \;or\; TD\; - \;1 \;treated \;group\; well}{Absorbance \;of\; Control\; well} \times 100. $$

### Determination of nitric oxide (NO)

RAW 264.7 cells were seeded in a 96-well microplate at 3 × 10^5^ cells/well with complete media. The cells were allowed grow and adhere for 24 h. The cells were treated with NQC (50, 25, 12.5, 6.25 and 3.125 µM) and negative control (0.1% v/v DMSO in water) for 4 h prior to LPS*Ec* (1 µg/mL) and allowed to incubate for 20 h at 37 °C, 5% CO_2_ incubator. Nitrite exist as a stable metabolite of nitric oxide, thus nitrite present in the supernatant was quantitatively measured as a chemical marker of nitric oxide (NO) production using Griess reagent (Promega, USA; 0.1% naphthylethylenediamine dihydrochloride and 1% sulfanilamide in 2.5% phosphoric acid). This assay was carried out following the manufacturer’s protocol where 100 µL of cell supernatant sample was incubated with 100 µL of Griess reagent at room temperature for 10 min and the absorbance at 540 nm was measured using Molecular Devices Spectramax M3 Multi-Mode microplate reader. A standard curve of sodium nitrite was plotted and used as a reference for extrapolation to quantify the amount of nitrite present.

### Determination of IL-1β, IL-6, PGE_2-_ and TNF-α

Production of IL-1β, IL-6, PGE_2−_ and TNF-α in RAW 264.7 cells were quantitatively measured using an enzyme-linked immunosorbent assay (ELISA). The cells were incubated with 0.1% DMSO or NQC for 4 h, then stimulated with LPS*Ec*, 1 µg/mL, for 20 h. The production of pro-inflammatory cytokines (IL-1β, IL-6) and TNF-α were determined using commercial ELISA kit (RayBio) according to the manufacturer’s instructions in product insert. The absorbance was measured at 540 nm. All the experiments were performed in triplicate.

### Nrf2 activation assay

The Nrf2 activation by NQC was determined using ‘Keap1:Nrf2 Inhibitor Screening Assay Kit’ (BPS Bioscience, USA) following the instructions in product insert. The NQC was dissolved in DMSO and diluted with assay buffer provided in the kit to produce the concentrations range, 100 µM to 1 nM. The fluorescence of the solutions in each well of 96-well plate was measured at λ_ex_ 485 nm and λ_em_ 530 nm. The dose–response curve was constructed plotting percent reduction in Kepa1-Nrf2 binding activity versus log concentration of NQC. From dose–response curve IC_50_ value was calculated.

### Molecular docking studies

To acquire molecular insights on the binding mode of NQC in Keap-1 binding site, molecular docking studies were carried out. Schrödinger small-molecule drug discovery suite 2018-2 was used to perform in silico docking studies. The crystal structure (PDB code: 5CGJ) used was sourced from ‘Research Collaboratory for Structural Bioinformatics Protein Data Bank’ (PDB) (http://www.pdb.org). The ‘protein preparation wizard’ was used to prepare the protein for molecular docking studies. This was followed by grid generation using ‘receptor grid generation wizard’ with default settings. The chemical structure of NQC was sketched in Maestro and prepared for docking using ‘ligprep’ wizard. Docking between the low energy conformation of NQC onto the binding site was performed by selecting the extra precision (XP) mode where protein–ligand structural motifs and water desolvation energy are incorporated to account for scoring function of binding free energy. The binding poses and affinity was further confirmed where “Induced Fit Docking’ (IFD) module was applied to NQC into the binding site. IFD module is docking protocol “Glide” and “Prime” where ligand flexibility and receptor flexibility are accounted for. GlideSP feature generates softened-potential docking protocol and was used to generate an initial number of 20 poses in the primary stage of IFD. Residues of the protein below 5.0 Å of ligand for each pose were refined. Docking of ligands from this is run again using GlideSP to generate poses. The top-ranked dock poses’ binding free energy (ΔG°) were calculated using the module Prime/Molecular Mechanics-Generalized Born Surface Area (MM-GBSA) with default settings.

### Metabolic stability assessment of NQC

A stock solution (SS) of NQC at 1 mM was prepared using 100% dimethyl sulfoxide. Working solution of 10 μM was further prepared by dilution of SS with 25% ACN and 50 mM tris–HCl buffer (pH 7.4). In a 96-well plate, NQC (effective concentration:1 µM) was incubated with phosphate buffer (pH7.4) and 0.5 mg/mL of liver microsomes (human, rat and mouse) at 37 °C in an incubation. Each NQC incubation with liver microsomes was performed in triplicates. The microsomal metabolic reaction was initiated by the addition of co-factor, NADPH (5 mM). Samples following the allotted time-point was drawn and quenched into a solution of acetonitrile containing 50 ng/mL internal standard. The sample was analysed by a developed HPLC method that has been validated for lower limit of quantification, linearity, precision, selectivity and accuracy according to FDA guidance [39].

Half-life of NQC was calculated using the equation:

$$ T_{1/2} = \frac{0.693}{K}. $$

The in vitro microsomal intrinsic clearance (CL_int_) was calculated based on the equation below:

$$ Cl_{int} = \left[ {\frac{0.693}{{in \;vitro\, {t_{(1/2)}} }}} \right] \times \left[ {\frac{{{\text{ml}}\; incubation}}{{{\text{mg}} \;microsome}}} \right] \times \left[ {\frac{{52.5\; {\text{mg}} \;microsome}}{{{\text{g}} \;liver}}} \right] \times \left[ {\frac{{52.5\; {\text{mg}}\; liver}}{{{\text{kg}}\; bw}}} \right]. $$

### Statistical analysis

The results were analysed and data are reported as mean ± standard deviation. The significance of the values was determined using student *t* test. Statistical significant difference was defined as P-value less than 0.05 (P < 0.05). All the statistical analyses were performed using Micosoft Excel.

## Results and discussion

### Characterisation of NQC

NQC was successfully synthesised and the chemical compound was spectroscopically elucidated using ^1^H Nuclear Magnetic Resonance (NMR), ^13^C Nuclear Magnetic Resonance, Fourier transform-infrared spectroscopy (FT-IR), and mass spectroscopy (MS). The overall schematic representation is as shown in Fig. 3. The target compound was synthesised and tested for purity by thin layer chromatography (TLC) using hexane and ethyl acetate (1:1) as the solvent system, and the R_f_ value calculated was 0.32. Structure of the compound was elucidated and confirmed using ^1^H NMR, ^13^C NMR, FT-IR and MS.

Pale brown solid; Yield 80%; mp 203 °C; FT-IR; *v*/cm^−1^ = 3311.78 (NH), 1643.35 (CO), 1579.70 (NH), 1535.34 (C=C); ^1^H NMR (600 MHz, DMSO-d_6_,): δ_ppm_ = 11.00 (s, 2H), 9.48 (d, 1H), 9.08 (1, *J* = 2.4 Hz, 1H), 8.74–8.73 (d, *J* = 6.0 Hz, 1H), 8.27–8.22 (q, 12.0, 6.0 Hz, 2H), 8.22–8.20 (q, *J* = 6.0 Hz, 2H), 7.55–7.53 (m, *J* = 6.0 Hz, 1H); ^13^C NMR (150 MHz, DMSO-d_6_): δ_ppm_ = 164.8, 161.4, 148.7, 148.1, 144.0, 143.8, 142.9, 140.3, 135.3, 131.9, 130.7, 130.3, 128.9, 125.1; MS (m/z) (%) = 293 (100), 294 (16.2), 295 (0.9), 296 (0.9). Analysis for C_15_H_11_N_5_O_2_ (293.29): calcd.: C, 61.43; H, 3.78; N, 23.88; O, 10.91%; found: C, 61.55; H, 3.75; N, 23.79; O, 10.95%.

### Cell viability

**Fig. 5:**
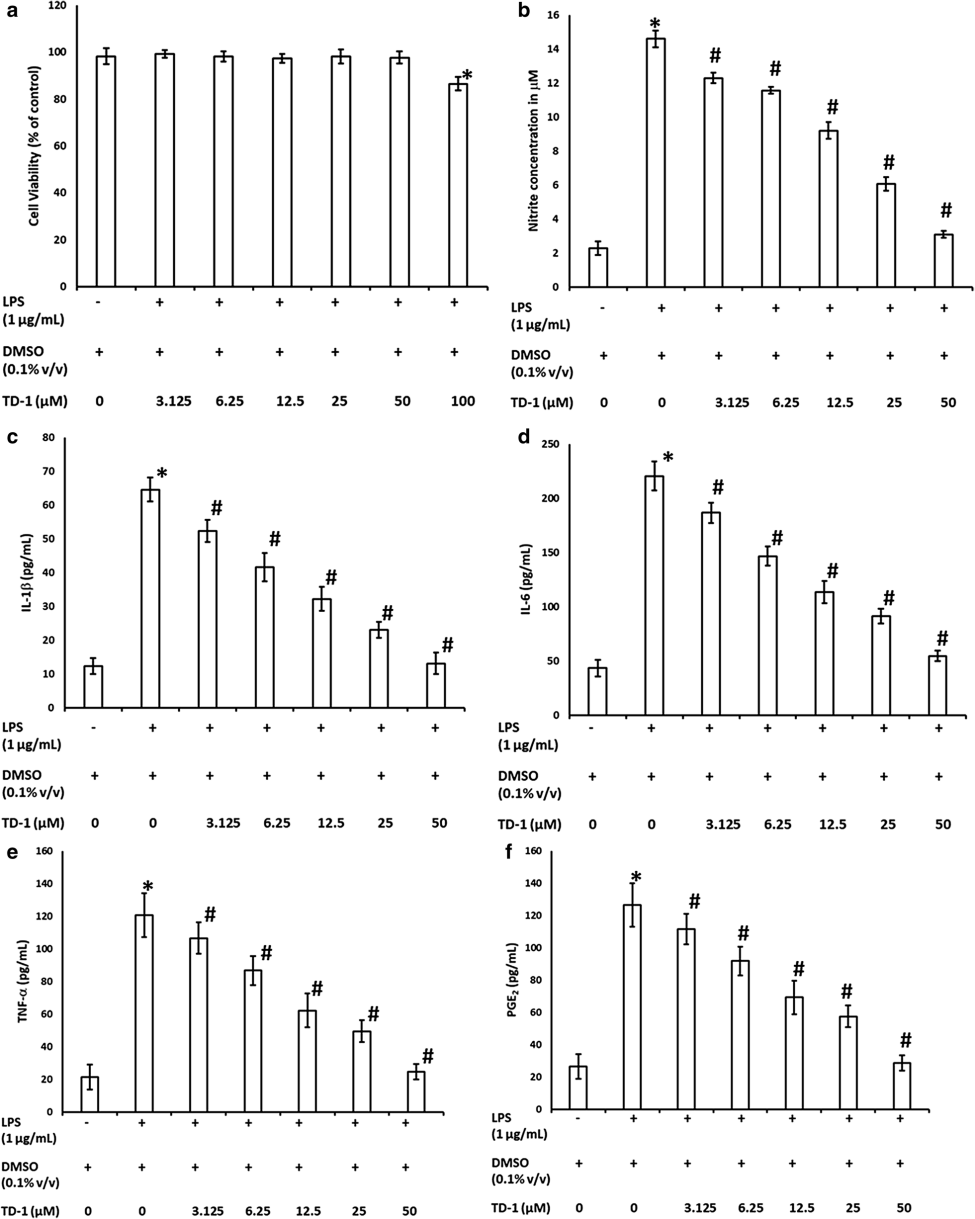
In vitro anti-inflammatory activity of TD-1 on RAW 264.7 cells. The effect of TD-1 on **a** cell viability; **b** LPS stimulated nitrite production; **c** LPS stimulated IL-1β production; **d** LPS stimulated 1L-6 production; **e** LPS stimulated TNF-α production and **f** LPS stimulated PGE_2_ production. In **a**; *Significant difference (P < 0.05) with respect to the cells (DMSO + LPS) treatment. In **b**–**f**; *Significant difference with respect to the cells (only DMSO treatment), ^#^Significant difference with respect to the cells (LPS + DMSO) treatment

The main purpose of this assay is to determine the NQC concentration range which is non-toxic to RAW 264.7 cells. The cytotoxicity effects of NQC (at a range of concentration) against RAW 264.7 cells stimulated with LPS*Ec* was performed using calorimetric MTT assay. Based on the results depicted in Fig. 5a, NQC do not show cytotoxic effects on RAW 264.7 cells at the tested concentrations of 3.125–50 µM (P > 0.05), while NQC at 100 µM shows significant toxicity (P < 0.05). Therefore, based on these results, NQC with concentrations between 3.125 and 50 µM was used for the subsequent experiments.

### Effect of NQC on NO production

NO production as an inflammatory mediator in the cell culture medium of LPS*Ec* stimulated RAW 264.7 cells is deduced using the Griess reaction [40]. The results are shown in Fig. 5b. The concentration of NO increased significantly in the LPS*Ec* stimulated RAW 264.7 cells (LPS*Ec* + DMSO) (P < 0.05) when compared to normal control (no LPS*Ec* + DMSO). This shows that NO production was induced by LPS*Ec* in RAW 264.7 cells. NQC between 3.125 and 50 µM significantly reversed the production of NO in LPS*Ec* induced RAW 264.7 cells in a dose-dependent manner (P < 0.05). It was reported that NO is involved in the regulation of multistage processes found in inflammation—particularly in the initial stages of inflammatory cells transmigrating to inflammation sites [41]. Therefore, NQC is found to be potent anti-inflammatory agent with an IC_50_ value of 26.13 ± 1.17 µM.

### Effect of NQC on pro-inflammatory cytokines (IL-1β, IL-6 and TNF-α) and PGE_2_

Inflammatory mediators and cytokines highly mediates inflammatory response especially in the initial stages of inflammation. These includes PGE_2_, NO; and pro-inflammatory cytokines namely IL-1β, IL-6 and TNF-α [42]. Subsequently, these cytokines will be responsible as upstream mediators where other inflammatory cytokines will be further stimulated and emitted, and ultimately leads to critical clinical symptoms of pain and its related immune disorders [43]. Therefore, the effects of NQC on inhibiting the production of IL-1β, IL-6, PGE_2-_ and TNF-α in the LPS*Ec* stimulated RAW 264.7 cells were quantitatively measured using ELISA. As depicted in Fig. 5c–f, the results from ELISA showed that the NQC in LPS*Ec* stimulated RAW 264.7 cells mediated the production of inflammatory cytokines and mediators at a dose-dependent manner compared to the negative control (P < 0.05) with IC_50_ values 13.27 ± 2.37, 10.13 ± 0.58, 14.41 ± 1.83 and 15.23 ± 0.91 µM respectively.

In the current study, it was found that NQC dramatically reduced the high levels of inflammatory mediators, NO radicals and PGE_2_, and pro-inflammatory cytokines of IL-1β, IL-6 and TNF-α stimulated by LPS*Ec* (Fig. 1b–f). In conclusion, it is believed that the NQC exerts desirable anti-inflammatory activity via suppression of excessive inflammatory mediators and inflammatory factors produced during inflammation.

### Effect of NQC on Nrf2 activation

Nrf2 is a transcription factor protein that contributes to the anti-inflammatory process by playing the role as an upstream regulator through binding with antioxidant response element (ARE); and is responsible in recruiting inflammatory cells and regulating gene expression. Anti-inflammatory gene expression and inhibition of inflammatory progression is regulated by Keap1/Nrf2/ARE signaling pathway. Under normal homeostasis conditions, Nrf2 remains ubiquitously bounded to the cytoskeletal protein Keap1. Due to Nrf2 activation, an anti-inflammatory response ensues. Thus, we determined the activity of NQC in inhibiting Nrf2-Keap1 interaction. The NQC showed dose-dependent activity in inhibiting Nrf2-Keap1interaction as shown in Fig. 2 and its IC_50_ value was 4.21 ± 0.89 µM.

### Molecular docking studies

**Fig. 6:**
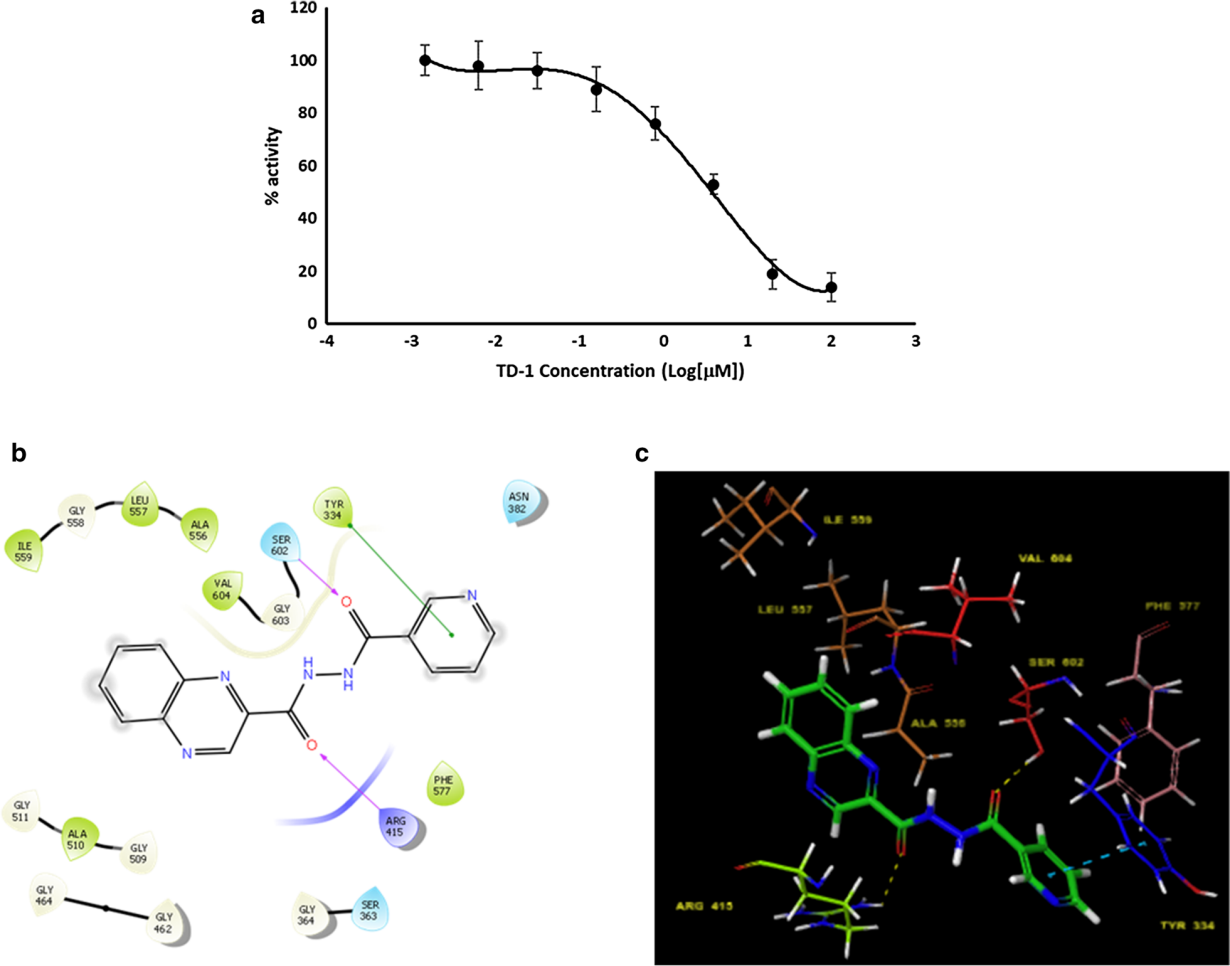
Nrf2 activation of TD-1. **a** The dose–response effect of TD-1 on inhibition of Nrf2/Keap1 interaction. **b** The 2D-intreraction diagram depicting H-bond, hydrophobic and pi-pi interactions between TD-1 and amino acid residues of the binding pocket of Nrf2/Keap1 interface (PDB ID: 5CGJ). The residues with light green shade denote the amino acids that form hydrophobic interactions with TD-1. **c** The 3D-interaction diagram showing interaction between TD-1 and key amino acid residues of binding pocket of 5CGJ

The primary docking of NQC with the binding pocket of 5CGJ was performed using Glide XP protocol where the flexibility of both ligand and receptor were disregarded. Here, favorable binding score of NQC at − 4.806 kcal/mol and ligand efficiency of − 0.218 kcal/mol were observed. Induced Fit Docking protocol was performed for NQC into the binding pocket where the introduction of residue flexibility is below 5 Å from the ligand. The IFD protocol presented highly negative IFD score (− 647.102 kcal/mol) indicating it is a favorable binding energy; and highly negative XP G score (− 7.051 kcal/mol). The binding free energy of the NQC was computed using MM-GBSA approach to further confirm it’s binding. The binding free energies of NQC was − 47.801 kcal/mol. The collective results give a confidence that NQC binds strongly in the binding site of Nrf2/Keap1 interface, which could be responsible for inhibition of Nrf2/Keap1 interaction. The 3D- and 2D-interaction diagram of NQC in the binding site of Nrf2/Keap1 interface is shown in Fig. 6b, c. As shown in the figures, the NQC forms hydrogen bond interactions with Ser 602 and Arg 415; hydrophobic interactions with Tyr 334, Ala 510, Ala 556, Ala 557, Ala 559, Phe 577 and Val 604.

### Metabolic stability studies

A calibration curve of peak area against the nominal concentrations of NQC was prepared using linear least-square regression model. The lower limit of quantitation (LLOQ) was determined to be 0.01 µM. The signal:noise ratio was found to be greater than 10. The linearity of the assay was r > 0.995 when assessed on the concentration range of 0.01–2.00 µM which is above the desired level of > 0.990. The assessment on intra- and inter-day precision and accuracy were carried out on three successive days using three concentration (0.02, 0.5 and 1.6 µM) where the intra- and inter-day precisions (RSD  %) was less than 9.00% and the accuracy (RE %) fell in the range of − 2.12 to 5.23%. The determination of NQC extraction recovery at three concentrations, 0.02, 0.5 and 1.6 µM was carried out and was achieved by comparing the peak area of extracted analyte in six replications (n = 6) with blank samples of post-extraction. The mean recovery was observed to be > 85.95%.

**Fig. 7:**
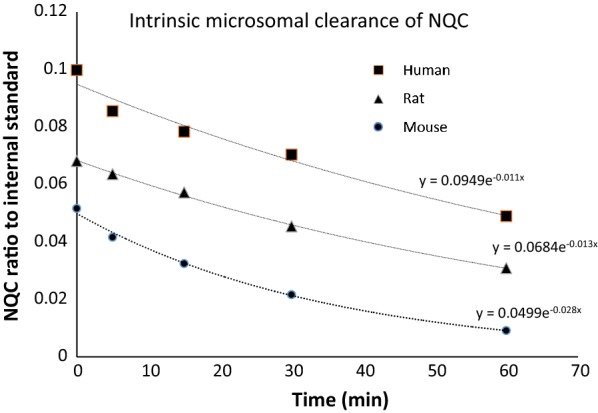
Intrinsic microsomal clearance of NQC in human, rat and mouse liver microsomes over time

In vitro microsomal metabolic stability assay using human, rat and mouse liver microsomes were performed to estimate half-life and microsomal intrinsic clearance of NQC at an effective concentration of 1 µM. The ratio of NQC to internal standard over time for human, rat and mouse liver microsomes are shown in Fig. 7 and the coefficient of correlations were more than 0.97. The observe in vitro T_1/2_ (63.30 ± 1.73, 52.33 ± 0.81 and 24.55 ± 0.34 min in human, rat and mouse respectively) as shown in Table 1, indicated that NQC presented faster clearance in mouse liver microsomes upon stimulation by the co-factor, NADPH. Whereas NQC presented moderate microsomal metabolism in rat and human liver microsomes. Correspondingly, the observed in vitro CL_int_ (1.14 ± 0.31, 1.39 ± 0.87 and 2.96 ± 0.34 µL/min/g liver in human, rat and mouse respectively) revealed that NQC displayed moderate stability (< 5 mL/min/g liver) in human, rat and mouse. From this in vitro assay, rat seems to have a closer value to human than that of mouse. From these findings, rat would be a suitable animal model for in vivo pharmacokinetics and metabolism of NQC in human.

**Table 1:** The microsomal intrinsic clearance of NQC of different species’ liver microsomes calculated based on the available data

Species of liver microsome	T_1/2_ (min)	mC_int_ (mL/min/g liver)	Rate of metabolism (min^−1^)
Human	63.30 ± 1.73	1.14 ± 0.31	0.011
Rat	52.23 ± 0.81	1.39 ± 0.87	0.013
Mouse	24.55 ± 1.13	2.96 ± 0.34	0.028

## Data Availability

All data generated or analysed during this study are included in this published article.

## Abbreviations

NRF2: nuclear factor erythroid-2-related factor 2
KEAP1: Kelch-like ECH-associated protein
NQC: *N*′-nicotinoylquinoxaline-2-carbohydrazide
LPSEc: lipopolysaccharide from *Escherichia coli*
IL: interleukin
TNF-α: tumor necrosis factor-α
PGE_2_: prostaglandin E_2_
IC_50_: inhibitory concentration, 50%
FBDD: fragment-based drug discovery
NO: nitric oxide
DMSO: dimethylsulfoxide
NADPH: reduced form of nicotinamde adenine dinucleotide phosphate
HLM: human liver microsomes
RLM: rat liver microsomes
MLM: mouse liver microsomes
DMEM: Dublecco’s modified eagle medium
FBS: fetal bovine serum
MTT: 3-(4,5-dimethylthiazol-2-yl)-2,5-diphenylterazolium bormide
PBS: phosphate buffer saline
ELISA: enzyme-linked immnosorbent assay
PDB: protein data bank
IFD: induced fit docking
SP: standard precision
MM-GBSA: molecular mechanics-generalized born surface area
HPLC: high performance liquid chromatography
FDA: food and drug administration
TLC: thin layer chromatography
NMR: nuclear magnetic resonance
MS: mass spectroscopy
FT-IR: Fourier transform infrared
